# Notes and News

**Published:** 1892-05-14

**Authors:** 


### Notes and News.

COLLECTED AND COMMUNICATED.

We are requested to state with reference to a paragraph which has appeared in the press upon the establishment of a Convalescent Home for Women, in which it was stated that "Mr. R. Frewer, of the Hospital Saturday Fund" had placed his services at the disposal of the Committee, that Mr. Frewer is not now in any way connected with the Hospital Saturday Fund, and that this organisation has nothing to do with the proposed Convalescent Home for Women. The Hospital Saturday Fund is already connected with twelve homes to which women can be sent.

The North London Hospital for Consumption will hold its annual festival on Saturday the 28th inst., at the Hotel Metropole and Mr. Henry Irving will preside. The Committee have commenced the much needed and long contemplated extensions of the institution. A freehold house has been secured in Fitzroy Square for the out-patient department, and the enlargement of the building at Hampstead to provide accommodation for thirty additional patients, with new quarters for the nursing staff, will be almost immediately commenced. The Committee have a portion of the necessary funds in hand, but they are in urgent need of further help.

Such an enterprise as the Industrial and Art Exhibition, held each year in the parish of St. Mark's, Battersea Rise, tends to counteract the tendency of the age towards superficiality, and an absence of excellence in any particular handiwork. This exhibition, although not strictly confined to the locality, is principally patronised by it. Since 1888 it has steadily increased in the excellence and number of exhibits, and we are glad to see that the plain needlework shown was especially excellent in the exhibition recently held. The articles were also greater in number than in any previous year. The entries are very low to encourage the humblest classes, and so excellent a scheme might be advantageously adopted in all large parishes or districts.

On Sunday, the 1st inst., the Dean of Worcester preached a sermon at St. Peter's, Cranley Gardens, in aid of the Brompton Consumption Hospital, and we are glad to see that the collection amounted to over £80. It is a most beneficial arrangement for all when congregations can thus have the work and needs of local charities brought before them from the pulpit. We often overlook many a deserving institution, which, if made known to us, we are only too glad to help.

The City of London Hospital for Diseases of the Chest, Victoria Park, will hold its annual festival at the Hotel Metropole on Tuesday, 24th inst., when the Right Hon. Earl of Strafford will preside. This hospital is in the heart of one of the most densely-populated districts of London, and needs and deserves the generous support of the public. At the present time the Committee are incurring a very heavy expenditure in redraining and renewing the sanitary arrangements of the hospital. This, therefore, is a time for new subscribers to put their shoulders to the wheel.

The West Ham Hospital held its annual festival dinner on the 4th inst., Mr. Courtenay Warner (the ex-High Sheriff of Essex) presiding. In proposing the toast of the evening, "Prosperity to the Hospital," the Chairman remarked that the institution was a great advantage to London, and to the East of London in particular, because of the relief which it gave to the London Hospital, already unable to bear the strain upon its resources. The West Ham Hospital was situated in the centre of a manufacturing district, where men were very liable to accidents from machinery, and although it was a small institution it was a very good one. It was hoped to add a new wing to the hospital before next year, and for this purpose £1,500 was required.

An interesting ceremony took place at the Royal Westminster Ophthalmic Hospital on the evening of the 4th inst., which derived additional importance from the fact that the date selected was the birthday of the founder, the late Mr. Guthrie, F.R.S. The occasion was to celebrate the opening of a new piano, largely subscribed for by Miss Guthrie, the founder's daughter, and friends of the hospital. The event excited a good deal of interest, and a large company present included Miss Ellen Terry, Mr. J. L. Toole, Sir Rutherford Alcock, K.C.B., Admiral Somerset, and other well-known personages and donors of the beautiful instrument, which was made by Broadwood and Sons. Lady Colin Campbell, who was unable to fulfil her promise to sing owing to indisposition, was present, and rendered assistance as accompanist to Miss Mendelssohn, who sang in her place, and whose singing was much appreciated. Miss Clara Sanderson (graduate of Trinity College) introduced the new piano to the audience in a most charming manner. Mrs. Moon Parker recited several humorous pieces by Oliver Wendell Holmes, and the proceedings terminated with a vote of thanks, proposed by Mr. Henry Power, F.R.C.S., Vice-president of the hospital, to Lady Campbell and the various performers for their able and efficient services, most freely rendered for the benefit of the patients, several of whom are purblind, and to whom, therefore, music is especially welcome.

